# Moral judgment modulation by disgust priming via altered fronto-temporal functional connectivity

**DOI:** 10.1038/s41598-017-11147-7

**Published:** 2017-09-07

**Authors:** Julian Lim, Yoanna A. Kurnianingsih, How Hwee Ong, O’Dhaniel A. Mullette-Gillman

**Affiliations:** 1 Center for Cognitive Neuroscience, Duke-NUS Medical School, 8 College Road, 169857 Singapore, Singapore; 2 Neuroscience and Behavioral Disorders Program, Duke-NUS Medical School, 8 College Road, Singapore, 169857 Singapore; 3 Department of Psychology, National University of Singapore, Block AS4, #02-07, 9 Arts Link, Singapore, 117570 Singapore

## Abstract

Moral judgments are not just the product of conscious reasoning, but also involve the integration of social and emotional information. Irrelevant disgust stimuli modulate moral judgments, with individual sensitivity determining the direction and size of effects across both hypothetical and incentive-compatible experimental designs. We investigated the neural circuitry underlying this modulation using fMRI in 19 individuals performing a moral judgment task with subliminal priming of disgust facial expressions. Our results indicate that individual changes in moral acceptability due to priming covaried with individual differences in activation within the dorsomedial prefrontal cortex (dmPFC). Further, whole-brain analyses identified changes in functional connectivity between the dmPFC and the temporal-parietal junction (TPJ). High sensitivity individuals showed enhanced functional connectivity between the TPJ and dmPFC, corresponding with deactivation in the dmPFC, and rating the moral dilemmas as more acceptable. Low sensitivity individuals showed the opposite pattern of results. Post-hoc, these findings replicated in the dorsal anterior cingulate cortex (daMCC), an adjacent region implicated in converting between objective and subjective valuation. This suggests a specific computational mechanism – that disgust stimuli modulate moral judgments by altering the integration of social information to determine the subjective valuation of the considered moral actions.

## Introduction

Decisions involve weighing the pros and cons of our available options, and in most cases comparisons are straightforward enough that a simple cost-benefit analysis can lead to a good decision. However, problems that contain a moral element are harder to resolve, due to difficulty in assigning values to outcomes such as harming someone else or even taking a life. This struggle is shown through fierce societal debates around such issues as societal safety nets and the death penalty. Such moral decisions are made by integrating information from a range of moral, ethical, and social sources, and go beyond simple models of economic decision making.

One of the factors that can affect moral decision making is emotional state. Disgust has a particularly intimate relationship with moral cognition^1, 2^, and there have been numerous demonstrations that both subliminal and conscious priming with disgust stimuli or disgust-inducing stimuli cause changes in moral judgments^3–7^. Such effects have been explained through dual-process models of decision making^8–10^, in which both rational and social-emotional components determine moral choice. We recently specified this effect, showing that the direction and degree of the modulation of moral judgments by disgust priming is dependent on the sensitivity of an individual to disgusting stimuli – that presentation of disgust facial expressions result in increased acceptability of utilitarian actions for individuals with high disgust sensitivity and reduced acceptability for those with low sensitivity^6^. Further, we have replicated and extended this finding by showing that this effect extends to incentive-compatible choices in a economic task (extending from the moral foundation of personal harm to honesty), in which the disgust primes resulted in increased likelihood of cheating behavior in participants with high sensitivity and decreased likelihood of cheating for those with low sensitivity^5^. Intriguingly, combining the data across these studies demonstrated that the function was indistinguishable across these tasks, suggesting that the same cognitive and neural mechanisms may be at play in both.

While many prior neuroimaging studies have investigated the numerous systems that contribute to decision making, less is known about how emotional stimuli modulate the neural processing of moral judgments. Deliberative reasoning engages a dorsal network including such regions as the dorsolateral prefrontal cortex (dlPFC), dorsomedial prefrontal cortex (dmPFC), and posterior parietal cortex (PPC)^11–14^. Social-emotional information is encoded in areas such as the dmPFC, ventrolateral prefrontal cortex (vlPFC), ventromedial prefrontal cortex (vmPFC), angular gyrus, precuneus, and the temporal-parietal junction (TPJ)^12, 15–17^. The dmPFC shows frequent engagement during decision making processes, including theory-of-mind, perspective-taking^18^, and judging moral dilemmas^9, 19^. In a recent study, we specifically found that a subregion of the dmPFC (the dorsal anterior midcingulate cortex, daMCC) contains the information necessary to perform the transformation from objective value (count) to subjective utility (worth)^11^. The TPJ appears to be specifically engaged during tasks that involve social cognitions^15^. Finally, a recent study suggests that moral information is integrated within valuative signals encoded within the ventromedial prefrontal cortex^20^.

**Figure 1:**
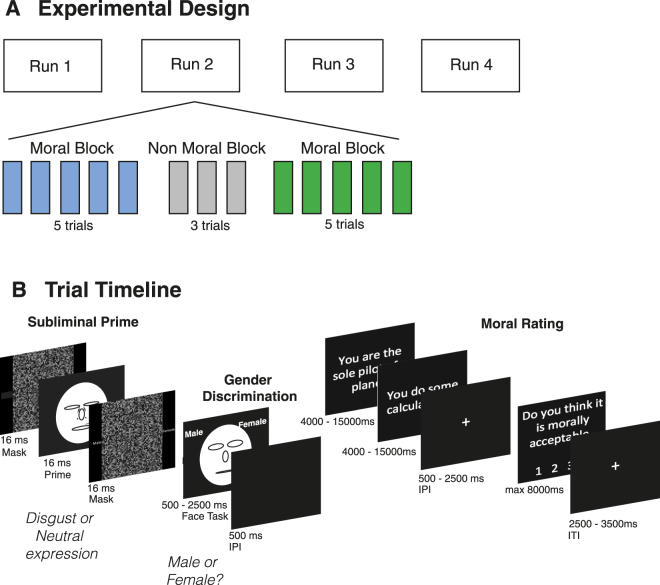
Task. (**A**) Subjects performed four task runs comprising 13 trials each. The first 5 and last 5 of these trials involved judgments of moral situations, while the central 3 involved judgments of non-moral situations. (**B**) Each trial consisted of a priming phase and a moral dilemma. In the priming phase, subjects were first exposed to a forward- and backward-masked face prime. Masks and primes were all presented for 16.66 ms (one monitor refresh at 60 Hz). They then had to rate a neutral target face as male or female (500–2500 ms). Moral dilemmas were presented in short vignettes split over 2 screens (15 s max each). With a 3^rd^ screen on which subjects rated how morally acceptable the action described in the vignette was on a 1 to 4 scale (8 s max). Please note, cartoon faces are used here to represent the actual human photographs used.

Our aim in the current study was to determine specifically how priming with disgust facial expressions alters the neural processing of moral judgment, and how individual differences in disgust sensitivity determine the degree and direction of modulation of moral judgment. Using fMRI, while participants performed the facial priming and moral judgement task (Fig. 1, see *Materials and Methods*), we uncovered a series of relationships relating altered moral judgment to changes in functional connectivity between the dmPFC and TPJ. These results provide a framework to explain the neural basis through which emotional and social information is integrated for moral decision making. In addition, post-hoc analyses indicate that the daMCC (a region adjacent to our dmPFC ROI which was recently found to contain the information necessary to transform from objective value to subjective utility), shows the same pattern of alterations – suggesting that the modulation of moral judgments may be due to alterations in the integration of social information in the transformation of value from objective to subjective representations (from count to worth).

## Results

### Gender discrimination accuracy

Accuracy on the gender discrimination task was high (93.1% (SD: 2.95%)), indicating that subjects were attending to the faces at the time of the prime presentation.

### Response times

There was no significant difference in moral judgment response times across priming conditions (neutral: mean = 3526 ms (SD: 814); disgust: mean = 3415 ms (SD: 937); within-subject t-test, t_18_ = −0.89, p = 0.39). Participants were significantly slower in responding to non-moral dilemmas (3914 ms (SD: 639)) than either moral dilemma condition (within-subject t-tests, disgust: t_18_ = −3.3, p = 0.004; neutral: t_18_ = −2.6, p = 0.02).

### Moral judgments

Acceptability ratings for moral dilemmas were coded on a 1–4 scale (1 = unacceptable; 2 = somewhat unacceptable; 3 = somewhat acceptable; 4 = acceptable). We note, again, that questions were phrased such that the participant was stating the acceptability of a utilitarian action (i.e., causing harm to one to save many).

**Figure 2:**
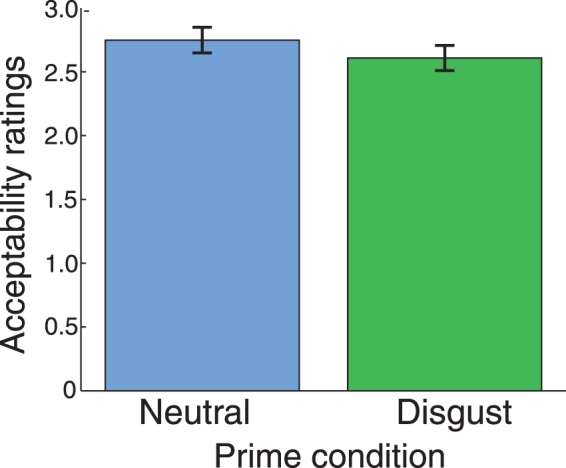
Behavioral data. On average, disgust priming caused subjects in this fMRI experiment to rate the utilitarian action in moral dilemmas as significantly less acceptable (t_18_ = −2.4, p = 0.03). Bars represent standard errors.

We found a significant effect of priming: on average, subjects rated utilitarian actions as being significantly less acceptable in the disgust than the neutral condition (Fig. 2; t_18_ = −2.4, p = 0.03). This demonstrates a clear effect of the priming manipulation on behavior, and is in the direction previously reported in multiple early studies^7, 21^.

In three previous behavioral experiments^5, 6^, we found that the average individual change in acceptability ratings due to emotional priming was correlated with individual differences in disgust sensitivity (DS-R). Within the current smaller fMRI sample, this relationship was not significant (r = 0.02, p = 0.94). We further investigated this non-significant result by conducting reliability analysis of the DS-R on our fMRI and behavioral samples. Cronbach’s alpha was 0.81 for our behavioral participants, but only 0.56 for the current fMRI sample. In comparison, Olatunji *et al*.^22^ reported a Cronbach’s alpha of 0.84 during scale development. Hence, it is possible that non-conscientious responding on the DS-R in this sample may have partly driven the failure to replicate the correlation between disgust sensitivity and the priming effect. Potential differences in task performance in the MRI scanner may also have played a role.

Critically, participants demonstrated significant modulation of moral acceptability due to the manipulation in the fMRI sample (on average). Specifically, the manipulation produced alterations in the moral judgments of individuals, and the individual variability in these changes can be leveraged to identify the neural structures that are responsible for this modulation.

### Contrasting neural processing of moral and non-moral decision making

**Figure 3:**
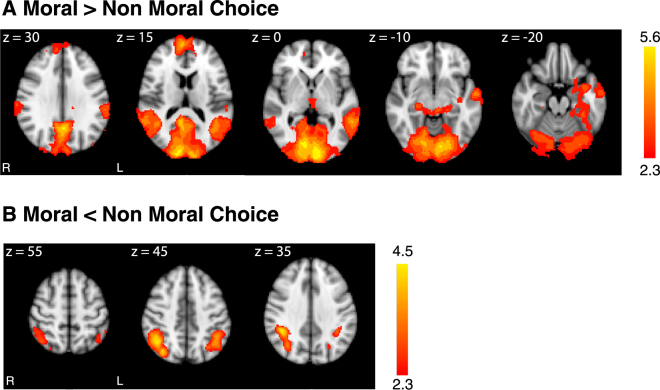
fMRI activation during moral and non-moral choices. (**A**) Increased responses to moral > non-moral choices were seen in large areas of anterior medial prefrontal cortex, precuneus, angular gyrus, middle temporal gyrus, superior temporal sulcus, and occipital cortex. (**B**) Increased responses to non-moral > moral choices were seen in right supramarginal gyrus, left intraparietal sulcus, and the superior parietal lobule bilaterally.

We investigated brain activation associated with moral decision-making by contrasting the decision phases of moral and non-moral trials. For the moral > non-moral contrast, we observed significant differences in large areas of frontal, temporal, and occipital cortex (Fig. 3A and Table 1). In the reverse direction (non-moral > moral), we found differences bilaterally in right supramarginal gyrus, left intraparietal sulcus, and the superior parietal lobule bilaterally (Fig. 3B and Table 1).

**Table 1:** Areas with significantly different activation (p < 0.05, whole-brain corrected) between moral and non-moral judgments.

Anatomical region	Number of voxels	MNI coordinates of local maxima			Z-score
		*X*	*Y*	*Z*	
Visual cortex	22031	−6	−94	0	5.61
		−8	−88	−4	5.58
		10	−98	8	5.52
		16	−78	−6	5.36
		18	−98	8	5.22
Precuneus		6	−54	20	5.26
L middle temporal gyrus	4582	−58	−52	8	5.04
		−62	−6	−12	4.21
L angular gyrus [temporal parietal junction]		−44	−52	16	4.27
L lateral occipital cortex		−36	−64	18	4.49
		−56	−66	6	4.25
R angular gyrus [temporal parietal junction]	2494	60	−50	16	3.97
R supramarginal gyrus		52	−40	18	3.71
R angular sulcus		54	−54	12	3.9
R superior temporal sulcus		52	−40	10	3.84
R middle temporal gyrus		46	−56	12	3.88
		42	−54	14	3.98
Frontal pole [anterior medial prefrontal cortex]	1904	6	60	12	4.71
Paracingulate gyrus		14	50	20	3.62
		−14	46	14	2.52
		12	50	2	2.81
R superior frontal gyrus		22	50	26	2.71
R supramarginal gyrus	2019	44	−44	36	4.55
R lateral occipital cortex		42	−58	46	4.37
		36	−70	44	4.12
L supramarginal gyrus	1134	−44	−48	44	3.63
L angular gyrus		−42	−58	44	3.61
L lateral occipital cortex		−30	−64	44	3.13
		−26	−68	38	2.88
L angular gyrus		−24	−58	30	2.52

Anatomical regions are designated by their Harvard-Oxford Atlas designation, with our common name included parenthetically.

### Contrasting neural processing of disgust and neutral priming – presentation and decision

As a preliminary analysis, we examined the contrast of the disgust and neutral conditions during the prime presentation and decision phase. No significant clusters were revealed by these whole-brain analyses.

### Relating variability in individual behavioral modulation to variability in differences in neural activation

**Figure 4:**
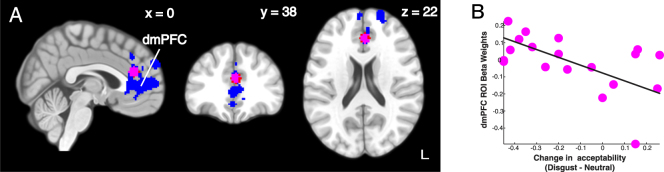
Neural correlates of change in moral acceptability. (**A**) Using behavioral modulation as a covariate during the disgust priming phase, we found significant negatively correlated signals in dorsomedial prefrontal cortex (dmPFC) and right middle temporal gyrus. The approximate centroid of the dmPFC cluster (X:0, Y:38, Z:22) was used to create a region-of-interest (ROI) to seed further connectivity analysis. (**B**) Within the dmPFC ROI, we extracted the beta weights for each participant to illustrate their individual change in moral acceptability due to disgust priming. With the negative correlation, the lower the dmPFC activation with disgust priming, the more acceptable the utilitarian moral dilemmas were judged.

Our principal goal, based on our prior behavioral studies^5, 6^, was to identify the brain regions whose activations were modulated as a function of the individual change in moral judgment due to the prime manipulation. To perform this individual differences analysis, we took the degree of behavioral change in moral acceptability ratings for each individual (the average acceptability ratings during the disgust blocks minus the neutral blocks) as our between-subject covariate (entered as a parametric covariate at the between-subject level of our FSL model). Analyses were limited to moral trials, examining the disgust or neutral primes during both the presentation and decision periods. During the presentation of the disgust prime, we found two large clusters of voxels that negatively correlated with behavioral modulation. The larger of these clusters (2327 voxels) extended from the frontal pole through medial rostral prefrontal cortex ventrally into the anterior cingulate cortex, while the smaller cluster (987 voxels) was localized to right middle and inferior temporal cortex (Fig. 4 and Table 2). No voxels showed significant positive relationships to the behavioral covariate during the neutral prime presentation or the decision periods for either the disgust- or neutral-primed trials. As our first step in analyzing these results, and with concern to avoid multiple comparison issues, we chose to focus further analyses on a sub-cluster of voxels within the dmPFC (illustrated in Fig. 4A, see *Methods*), based on prior studies that have shown the importance of this area in decision making.

**Table 2:** Peaks of activation during the disgust prime using change in moral judgment (disgust – neutral) as a between-subject covariate.

Anatomical region	Number of voxels	MNI coordinates of local maxima			Z-score
		***X***	***Y***	***Z***	
R frontal pole	2327	12	48	42	3.73
		16	68	28	3.28
Frontal pole [anterior medial prefrontal cortex]		6	62	28	3.59
Paracingulate gyrus [ventromedial prefrontal cortex]		−2	42	4	3.52
		8	44	0	3.38
		−2	42	4	3.27
R middle temporal gyrus	987	66	−30	−10	3.99
		58	−8	−30	3.03
R inferior temporal gyrus		58	−2	−38	3.34
		60	−12	−32	3.02
		56	−16	−30	2.86
		48	−6	−34	2.73

Anatomical regions are designated by their Harvard-Oxford Atlas designation, with our common name included parenthetically.

### Alterations of functional connectivity by manipulation

**Figure 5:**
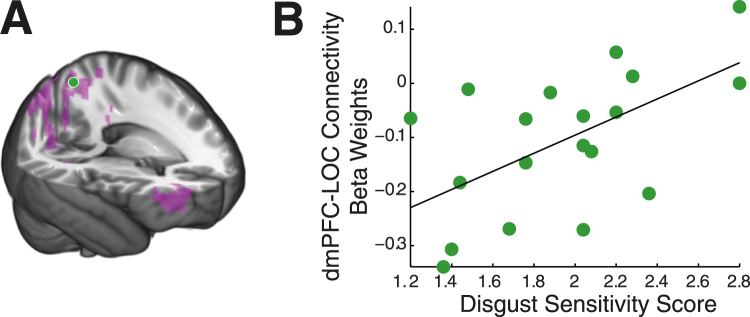
dmPFC functional connectivity during disgust prime presentation. (**A**) Significant functional connectivity (negative) was seen between dorsomedial prefrontal cortex (dmPFC) and posterior cingulate cortex, right superior frontal gyrus, and lateral occipital cortex (LOC) bilaterally. (**B**) Connectivity strength between dmPFC and left LOC is significantly correlated with individual disgust sensitivity.

**Figure 6:**
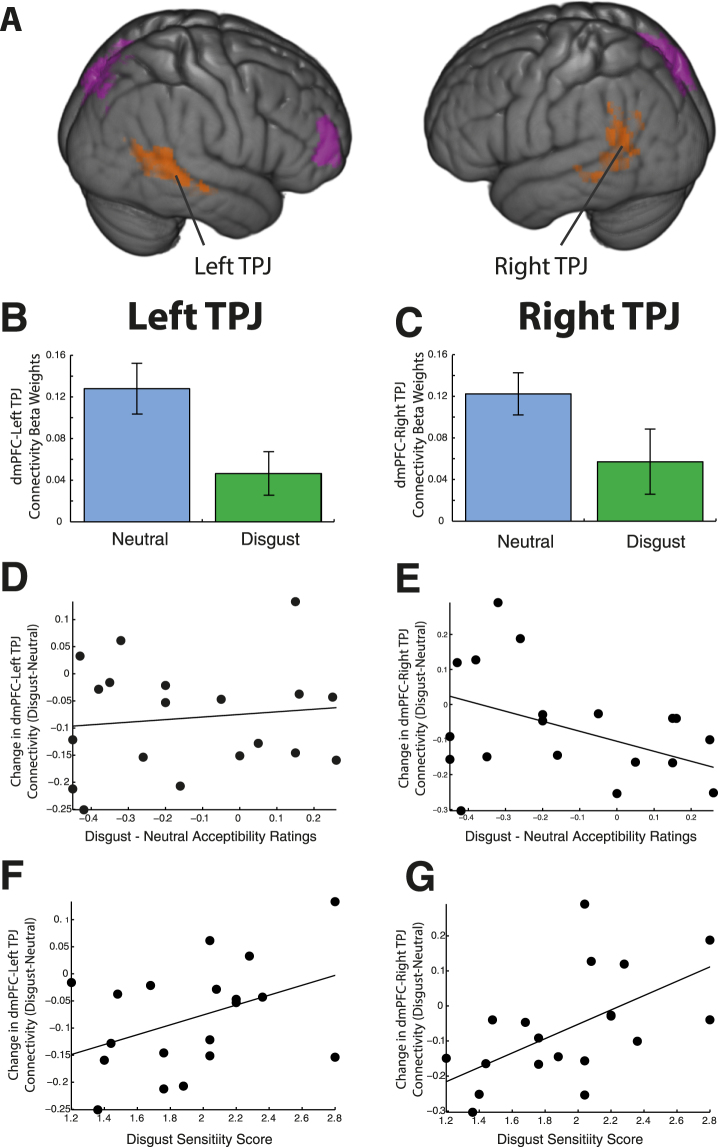
dmPFC functional connectivity: dynamic connectivity by prime. (**A**) Areas of negative functional connectivity with dorsomedial prefrontal cortex (dmPFC) are similar during the disgust and neutral prime. However, significant positive connectivity was observed with bilateral temporal-parietal junction (TPJ) only during the neutral prime. (**B**,**C**) Connectivity weights are significantly higher in bilateral TPJ during the neutral than the disgust prime. Bars represent standard errors. (**D**,**E**) Relation between change in dmPFC-TPJ connectivity across primes and modulation in moral judgment, resulting in a significant negative correlation in right TPJ. (**F**,**G**) Changes in dmPFC-TPJ connectivity between states are correlated with individual disgust sensitivity scores.

Using the dmPFC ROI, we tested whether changes in functional connectivity between this ROI and other brain regions could produce a viable mechanism that might account for the individual changes in activation in this region (and ostensibly the prime-induced behavioral changes). We used a psychophysiological interaction (PPI) model to identify voxels with functional connectivity to our dmPFC ROI at the time of either disgust or neutral priming (separately). During disgust priming, this ROI showed significant negative connectivity with a large cluster that included the posterior cingulate cortex, precuneus, right superior frontal gyrus, and lateral occipital cortex (LOC) bilaterally (Fig. 5). Interestingly, during neutral priming, we observed highly similar patterns of negative connectivity with the addition of significant positive connectivity in the temporal-parietal junction (TPJ) bilaterally (Fig. 6 and Table 3).

**Table 3:** Peaks of positive and negative connectivity during the disgust and neutral primes using dmPFC as a seed region.

**Anatomical region**	**Number of voxels**	**MNI coordinates of local maxima**			**Z-score**
		***X***	***Y***	***Z***	
***Disgust prime (positive connectivity)***					
No voxels found					
***Disgust prime (negative connectivity)***					
L lateral occipital cortex	7234	−24	−68	50	3.53
R lateral occipital cortex		34	−64	34	3.46
Posterior cingulate gyrus		4	−28	42	3.45
Precuneus		14	−72	42	3.45
Precentral gyrus		8	−30	50	3.38
		14	−22	46	3.33
R frontal pole	888	50	50	6	3.58
		28	44	4	3.52
		46	58	6	3.45
		48	54	4	3.37
		36	58	10	3.36
		36	60	14	3.28
***Neutral prime (positive connectivity)***					
R middle temporal gyrus	832	66	−50	8	3.48
		64	−60	8	3.39
		72	−38	4	3.3
		64	−32	−6	2.93
R angular gyrus [temporal parietal junction]		50	−52	16	3.12
R lateral occipital cortex		60	−64	14	3.09
L lateral occipital cortex	765	−58	−62	24	3.68
L middle temporal gyrus		−58	−58	6	3.1
		−62	−54	10	2.99
		−66	−48	0	2.85
L superior temporal gyrus		−60	−38	8	2.96
L angular gyrus [temporal parietal junction]		−48	−60	22	2.9
***Neutral prime (negative connectivity)***					
Precuneus	3958	4	−80	50	4.01
		6	−74	56	3.91
		−2	−82	48	3.88
R lateral occipital cortex		10	−84	44	3.95
		8	−82	48	3.92
		−8	−86	42	3.89
Posterior cingulate gyrus	2696	2	−38	18	4.2
		8	−38	24	3.92
		0	−44	2	3.78
		4	−38	8	3.68
		6	−40	34	3.48
		22	10	34	3.73
R frontal pole	788	48	56	4	3.71
		38	52	20	3.67
		28	46	16	3.25
		36	62	12	2.64

Anatomical regions are designated by their Harvard-Oxford Atlas designation, with our common name included parenthetically.

We explicitly tested for changes in functional connectivity by constructing an ROI for each of the bilateral TPJ clusters, and extracting the beta weights of the connectivity with the dmPFC ROI in each condition. These TPJ-dmPFC connectivity beta weights differed significantly between the disgust and neutral conditions in the left hemisphere (t_18_ = 3.54, p = 0.002), and showed a trend to significance in the right (t_18_ = 1.82, p = 0.08) (Fig. 6B,C).

Further, we tested whether the individual change in functional connectivity between the dmPFC and these TPJ ROIs could account for the individual change in moral judgment, and found a significant correlation for the right TPJ (r = −0.31, p = 0.01), but not the left (r = 0.10, p = 0.68) (Fig. 6D,E).

Finally, given our previously found relationship between individual disgust sensitivity (DS-R scores) and individual modulation of moral judgment^6^, we investigated whether individual differences in TPJ-dmPFC connectivity (disgust minus neutral) might be correlated with individual DS-R scores (which we had purposefully not utilized in prior analyses within this experiment). We found evidence that this was the case (Left: r = 0.40, p = 0.09; Right: r = 0.55, p = 0.01) (Fig. 6F,G).

We tested the specificity of these functional connectivity results by replicating the analyses within the identified LOC cluster, a region which showed significant functional connectivity with the dmPFC in both conditions but is not considered to be involved in decision making. We constructed an ROI at the peak absolute functional connectivity in the right LOC cluster, and extracted the average beta weights within the ROI for each participant. In contrast with the TPJ, there was no change in LOC-dmPFC connectivity between conditions (t_18_ = 1.24, p = 0.23). Like the TPJ, individual LOC-dmPFC functional connectivity correlated with individual DS-R scores (Fig. 5B) (Disgust: r = 0.55, p = 0.01; Neutral: r = 0.41, p = 0.08), but without significant change in these correlations across the conditions (z = 0.51, p = 0.61). In other words, the LOC-dmPFC functional connectivity is stable across the manipulation while still showing a relationship between functional connectivity and individual disgust sensitivity.

### Post-hoc replication of the functional connectivity analyses using alternative daMCC ROI

In a recent study, examining economic decision making, the daMCC was shown to contain the interaction of parametric value signals and individual preferences necessary for the transformation from objective value (count) to subjective utility (worth)^11^. As the dmPFC ROI we identified in this experiment is adjacent to this daMCC region, we sought to examine whether the pattern of results we found with the dmPFC ROI replicated within the daMCC – the negative relation of activation to change in moral acceptability and the alterations of functional connectivity with the TPJ. If so, this would suggest a potential computational mechanism through which disgust stimuli modulate moral judgment.

**Figure 7:**
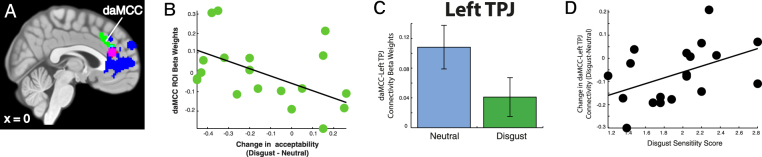
Replication of the functional connectivity analyses in the daMCC. (**A**) The location of the daMCC ROI in relationship to the constructed dmPFC ROI (X:0, Y:38, Z:22). The same patterns of results were found using this alternative ROI: (**B**) the beta weights extracted from the daMCC shows the same negative correlation with individual change in moral acceptability, (**C**) connectivity weights are still significantly higher in bilateral TPJ during the neutral than the disgust prime, and (**D**) changes in daMCC-TPJ connectivity between conditions are also correlated with individual disgust sensitivity scores.

The pattern of results replicated (Fig. 7). First, we tested whether individual activation levels in this daMCC region could account for individual change in moral acceptability. We found that individual daMCC ROI beta weights were marginally significantly correlated to change in moral acceptability ratings (r = −0.41, p = 0.08). Secondly, we replicated the functional connectivity analysis using the daMCC ROI, and extracted beta weights from the left TPJ cluster for both the disgust and neutral conditions. Mirroring the results of the adjacent dmPFC region, in the neutral condition we found significant positive functional connectivity between the daMCC and the TPJ ROI (Left: *t*_18_ = 3.80, p = 0.001), which was statistically absent in the disgust condition (Left: *t*_18_ = 1.61, p = 0.12). The within-subject change in connectivity between the daMCC and left TPJ were significant between the disgust and neutral conditions (*t*_18_ = 2.37, p = 0.03). As the final component of the analysis set, we tested whether individual disgust sensitivity could account for the change in the left TPJ-daMCC connectivity between conditions. We found a marginally significant correlation between the connectivity beta weight and individual disgust sensitivity score (r = 0.44, p = 0.06). These results show that the pattern of results obtained with the dmPFC ROI generalize to an adjacent region (daMCC).

### Results Summary

Utilizing a seed ROI derived from the voxels whose activations across individuals had been shown to covary with individual changes in moral judgment, we found significant functional connectivity with multiple brain regions. Contrasting between the neutral and disgust conditions, we saw a suggestion of priming-driven changes in functional connectivity between the dmPFC ROI and bilateral TPJ clusters. Explicitly testing, we found this difference to be significant. Further, this individual change in TPJ-dmPFC functional connectivity was significantly correlated to the individual change in moral judgment (for the right TPJ cluster only). Finally, this change in TPJ-dmPFC functional connectivity was significantly correlated to the individual trait of disgust sensitivity (which we had purposely not included in prior analyses, but which we had found in prior behavioral experiments to predict the individual degree of change in moral judgment due to priming^5, 6^). These results were replicated in an adjacent region, the daMCC, which has been specifically implicated in the transformation from objective value to subjective value, providing a potential computation whose modulation may account for the modulation of moral judgments by emotional stimuli.

## Discussion

**Figure 8:**
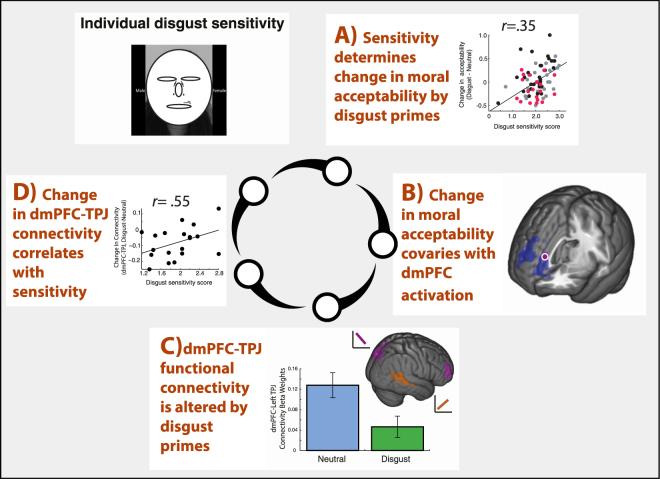
Disgust priming and moral judgment: bridging brain and behavior. Combined, our results suggest a model describing a closed circle of relationships that govern the effects of disgust priming on moral judgment. (**A**) Individual disgust sensitivity modulates the effect of disgust priming on the acceptability of utilitarian actions in personal-harm moral dilemmas. (**B**) The degree of this modulation covaries with activation in the dorsomedial prefrontal cortex (dmPFC). (**C**) During neutral priming, the dmPFC is functionally connected with bilateral temporal-parietal junction (TPJ), with absent connectivity (significantly reduced) with disgust priming. (**D**) The individual difference in connectivity strength between dmPFC and TPJ between the disgust and neutral conditions correlates with individual differences in disgust sensitivity, mirroring the relationship between disgust sensitivity and the final changes in moral acceptability. Parsimoniously, these results suggest that, in an individual with high disgust sensitivity, the disgust primes enhance connectivity between the dmPFC and TPJ, resulting in decreased activation in the dmPFC and greater acceptability ratings of utilitarian actions in the moral dilemmas.

We examined the neural mechanisms that underlie changes in moral judgment due to presentation of disgust stimuli (disgust priming). A theoretical model summarizing our key findings is presented in Fig. 8. In short: (A) disgust priming modulates moral judgments based upon individual sensitivity to disgust. (B) The individual degree of change in moral judgment is neurally represented, at the time of disgust priming, by individual activation levels in the medial frontal and right middle temporal cortices. (C) Focusing on the medial frontal cluster, the dmPFC ROI has prime-dependent functional connectivity with bilateral superior temporal sulcus (STS)/TPJ clusters. (D) Individual prime-dependent change in functional connectivity between the dmPFC and bilateral STS/TPJ clusters is correlated with individual disgust sensitivity. We discuss each of these components in turn, before considering the significance of our proposed model as a whole.

### Brain activation during moral vs. non-moral dilemmas

In a seminal study, Greene *et al*.^9^ found that moral-personal dilemmas (such as those in the current study, with imagined direct contact with the victim) produced greater activation within the medial frontal gyrus, angular gyrus, and posterior cingulate gyrus compared with moral-impersonal and non-moral dilemmas (also shown in ref. 19). We replicate these findings in our current dataset, showing greater activations to moral dilemmas (personal) compared to non-moral dilemmas within highly similar regions.

### Individual activation in dmPFC to disgust primes covaries with modulation of moral judgment

Behaviorally, we found clear modulation of moral judgment across individuals. Taking advantage of individual differences, we performed a covariate analysis to identify brain regions in which individual activation to disgust priming covaried with individual differences in moral judgment between the neutral and disgust conditions. This identified a large stretch of anterior frontal cortex, including the dmPFC, which is generally associated with decision making, reasoning, and executive functions^11–13^, and related specifically to moral evaluation in a recent ALE meta-analysis^23^.

The dmPFC has also been implicated in tasks requiring social cognition (for reviews see refs 24–26), and specifically with processes such as mentalizing (or theory-of-mind)^27, 28^. Abnormal dmPFC activity has been reported in autistic spectrum disorder patients^29^, a condition strongly associated with deficits in theory-of-mind processes. Utilizing Neurosynth^30^ (neurosynth.org, retrieved April 2017), we examined the posterior probabilities of cognitive functions being engaged given activation in the centroid of our dmPFC ROI (X:0, Y:38, Z:22). Concurring with our literature review, keywords with high posterior probabilities were relevant to decision making in general (e.g. “executive functions” (0.75) and “preferences” (0.75)), and social and moral decision making specifically (e.g. “social interaction” (0.78), moral (0.73)).

Interestingly, it was recently shown that dmPFC microstate fluctuations, prior to the presentation of available options, could predict what option a participant would later select^31^. This suggests that within-subject fluctuations in dmPFC activations can bias future choices, concurring with our between-subject finding that activations covary with individual change in behavioral moral acceptability.

Our current findings extend knowledge of the function of the medial prefrontal region by suggesting that it may be the brain module that performs the final integration of socio-emotional and quantifiable information in moral decision making. Across decision making domains, this may be a general function of the dmPFC.

### dmPFC is functionally connected to STS/TPJ in neutral but not disgust primed trials

Functional connectivity analyses revealed prime-dependent connectivity between the dmPFC and TPJ. These regions were functionally connected during neutral trials, but not during disgust trials, with a significant reduction between these two conditions. This pattern of prime-dependent alteration of functional connectivity was found bilaterally (more strongly in the left hemisphere).

The TPJ is uniquely involved in processing information regarding other agents whose behavior is relevant to one’s own choices^15^. Temporary down regulation of the right TPJ through transcranial magnetic stimulation reduces participants’ dependence on information about an agent’s mental state during harm judgments^32^. Majdandzic and colleagues^33^ demonstrated enhanced connectivity between the TPJ and anterior cingulate regions when subjects were asked to take the perspective of the victim when considering trolley-car type dilemmas.

We infer from our functional connectivity analysis that disgust primes modulate the efficiency by which information processed in the TPJ is communicated to the dmPFC. In contrast, functional connectivity between dmPFC and LOC was not modulated by our priming manipulation, indicating the specificity of the altered functional connectivity between the dmPFC to TPJ, and that this is not a general effect of the priming. Combined, the manipulation-dependent altered functional connectivity between the TPJ and dmPFC suggests the reverse inference that our effects are occurring through the modulation of the integration of social information in the decision making process.

### Change in dmPFC-TPJ connectivity is correlated with individual disgust sensitivity

Alterations in dmPFC-TPJ connectivity were correlated with individual sensitivity to disgust. Critically, this variable was not entered into our fMRI model. Inspection of Fig. 6F,G indicate that the strength of dmPFC-TPJ connectivity obeys a similar bidirectional function to that found in our behavioral experiments^6^. Furthermore, the change in connectivity strength between the dmPFC and right TPJ was also correlated with the modulation of moral judgment by disgust primes.

Our results suggest a neural model through which moral judgment depends upon the integration of social information, and that the degree of this integration is modulated by individual sensitivity to emotional information. The observation that right TPJ connectivity change correlates both with disgust sensitivity and modulation in moral judgment suggests a particularly important role for this lateralized region, and is in agreement with previous studies that attribute theory-of-mind function specifically to right TPJ^34, 35^. We posit that individuals with high sensitivity to disgust have increased connectivity between the dmPFC and TPJ, which leads to decreased activity in dmPFC and increased acceptability of the utilitarian actions described in our vignettes. This formulation is in line with a recent finding pointing to the independent encoding of utilitarian judgments in these two areas^20^. However, as PPI analyses do not contain information about the direction of the found functional connectivity, this hypothesis remains to be tested.

Intriguingly, using the reverse inference that TPJ signals social information, these results suggest that enhanced involvement of social information results in greater acceptability of utilitarian actions. This is in opposition to the philosophical view that social information lies in the societal rules (e.g. “thou shalt not kill”) that dictate deontological judgments. In essence, while utilitarian judgments could be considered as focusing on the numbers without considering the people, these results suggest humanistic neural processing - the fact that these numbers *are* people underlies how they are processed in the brain. We note that this inference could not have been made based on behavioral results alone.

### Post-hoc replication to the daMCC – modulation of the transformation to subjective worth?

The above results suggest that disgust stimuli alter the integration of social information in the judgment of moral acceptability, but they do not provide for a specific mechanism. In a recent study, it was shown that the daMCC contains the information necessary to perform the transformation from objective to subjective value – from count to worth. This was done using a monetary risky decision making task. As this daMCC region was directly adjacent to (but non-overlapping with) the dmPFC ROI found in this study, we sought to test the robustness of our findings across moral and economic domains, and to test for a potential mechanism through which the disgust primes may modulate moral acceptability. We found a complete replication – the daMCC activation was related to the change in moral judgment, the daMCC ROI was functionally connected to the TPJ during neutral trials and significantly less connected during disgust trials (to non-significant connection), and the degree of change in connectivity between the daMCC and TPJ was correlated to individual disgust sensitivity.

This replication suggests a potential mechanism through which disgust stimuli modulate moral judgment as moderated by individual sensitivity. In our prior study^11^, we found that daMCC activation was negatively related to the direction and degree of individual subjective worth for the offered gamble – that activation was associated with reduced subjective, deactivation with enhanced subjective valuation, and non-modulated activation was associated with non-modulated subjective valuation. In the current study, we see a similar pattern of results in the relationship between daMCC activation and the change in moral acceptability due to disgust facial expressions (Fig. 8). Activation of daMCC occurs with reduced acceptability, deactivation with enhanced acceptability, and non-modulation of activation falls close to non-modulation of acceptability.

These results suggest that the daMCC has the same functional role across both economic and moral domains – integrating contextual information to determine the transformation from objective count to subjective utility. By extension, disgust stimuli may modulate moral judgments by altering the computation of the subjective valuation of actions under consideration.

### A model of moral decision making – integration of social information modulated by emotional input

Combined, we reveal a dynamic neural system that may account for how moral judgment is modulated by emotional primes – a circle of relationships that bridge across brain, personality, and behavior. Our results suggest that social information is differentially integrated in the decision-making process based on individual sensitivity, as reflected by the degree of connectivity of dmPFC with TPJ (and daMCC with TPJ). However, TPJ activity on its own does not reflect the subsequent alteration of moral judgments. This alteration is only correlated with the level of activity in the medial frontal regions (dmPFC and daMCC) and the change in functional connectivity between the TPJ and dmPFC, suggesting that dmPFC is integral for the decision computations that determine one’s final choice.

Intriguingly, the dmPFC and TPJ have also been implicated in the deficits associated with autism spectrum disorders^29, 36^. Such patients also show different patterns of moral reasoning, driven both by reduced sensitivity to the feelings of others (impaired theory-of-mind) and a deficit in introspecting on emotions in the self^37^. The fMRI correlates of these differences have been localized to the dmPFC and TPJ^38^. We speculate that low sensitivity to emotional stimuli may be an analog to this trait, as healthy individuals weigh socio-emotional information in morally ambiguous situations.

In summary, our data indicate that disgust priming exerts its influence on moral decision-making via a frontal-temporal circuit with differential functional connectivity across prime conditions, dependent on individual sensitivity to the primes. Future research in this area could profitably investigate the behavioral and neural specificity of this effect – across emotional, stimulus, and moral categories.

## Materials and Methods

19 undergraduates (9 male, mean age = 22.63 (SD = 1.30)) were recruited from the National University of Singapore via advertising or word-of-mouth as participants in this experiment. Participants were right-handed with no history of chronic physical or psychiatric disorders. They were asked to refrain from consuming caffeine or medication 6 hours prior to the start of the experiment. Functional MRI (fMRI) scanning was conducted in the Duke-NUS Medical School. Participants provided written informed consent before engaging in the study, and were compensated SGD $40 for their participation. Approval to conduct this research was granted by the Institutional Review Board of the National University of Singapore. All methods were carried out in accordance to the approved guidelines.

Subjects underwent four runs of fMRI scanning (see *Task Description* below). Reading and responding to the dilemmas was self-paced with an upper limit on response time for each trial. Run length was fixed at 7 m 22 s, resulting in a variable period of fixation/baseline at the end of each run. In a very small number of runs, participants failed to complete all trials in the allotted time (2 trials total, from 2 different participants).

Following the fMRI scan, subjects filled out a number of questionnaires, including demographics and the Disgust Scale - Revised (DS-R^39^; modified by^22^), a short self-report survey of the participants sensitivity to disgust-related stimuli or scenarios. During debrief, participants were first asked to guess the purpose of the experiment – none guessed close to our purpose. Each participant was explicitly asked if they saw the subliminal primes – none reported awareness. Finally, as the protocol featured deceit through the use of subliminal primes, each participant was asked if they would like for us to remove their data from our study – none opted for removal of their data.

### Task description

We used a moral decision making paradigm (Fig. 1) in which moral acceptability judgments have been found to be reliably altered as a function of presentation of disgust primes and individual sensitivity to disgust stimuli^6^. Data were presented via E-Prime 2.0^40^. Participants were instructed that they would perform two alternating tasks: a gender discrimination task, and a moral decision making task. Unbeknownst to participants, the gender discrimination task was a vehicle for the presentation of subliminal facial primes.

For the gender discrimination task, each trial began with subliminal presentation of a facial prime (16 ms), with forward and backward masking (16 ms each). Immediately following, the same model’s face was presented with a neutral expression, and participants had 5 s to judge whether the model was male or female. Priming faces were of either disgust or neutral emotional expressions. Faces were drawn from the Karolinska Directed Emotional Faces (KDEF) database^41^, and adapted as described in our previous work^6^. An equal number of male and female faces were presented over the course of the paradigm, and models were not repeated.

For the moral decision making task, participants were presented with a vignette split over two consecutive screens, followed by a third screen containing a moral (M) or non-moral (NM) decision. This decision required subjects to rate whether the action presented in the vignette was ‘unacceptable’, ‘somewhat unacceptable’, ‘somewhat acceptable’, or ‘acceptable’. The task was self-paced with wide constraints. To encourage subjects to read the full vignette text, they could not proceed past each of the first two screens until at least 4 s had elapsed. Trials timed-out if a participant did not finish reading a screen within 15 s. Subjects were given a maximum of 8 s to rate the acceptability of the moral action described.

In each task run, subjects were presented with thirteen dilemmas in three blocks, consisting of five moral dilemmas, three non-moral dilemmas, and five more moral dilemmas. Moral dilemmas were ‘trolley-car type’ scenarios in which personal harm (injury or death) is inflicted on one individual in order to save the lives of several others. We previously developed and normed these dilemmas^6^, as a combination of prior studies^9^ and new items. Non-moral dilemmas consisted of everyday choices without moral implications (e.g., judging whether it is acceptable to travel to a destination by a quicker route rather than a more scenic, or whether it is acceptable to replace the type of nuts called for in a cooking recipe). The central period containing the non-moral dilemmas served as a “washout” period between the disgust and neutral priming trials.

Subjects were primed with disgust or neutral faces in the moral blocks in counterbalanced order, and with neutral faces in the non-moral blocks. Each run contained one disgust and one neutral block. A schematic of the task paradigm can be found in Fig. 1.

### Image acquisition

MR images were acquired on a 3 T Tim Trio system (Siemens, Erlangen, Germany). Visual stimuli were back-projected (Epsom EMP1715, 800 × 600 pixels, 60 Hz) onto a screen positioned behind the scanner bore. Subjects responded using the four fingers of their right hand on a custom-made four-button response box. Four runs of 206 volumes each were acquired using a gradient echo-planar imaging (EPI) sequence with the following parameters: repetition time (TR) = 2 s; echo time = 30 ms; flip angle = 90 degrees; field-of-view = 192 × 192 mm; matrix size, 64 × 64). Each volume consisted of thirty-six 3 mm axial slices aligned to the intercommisural plane. To aid with registration into standard space, we also obtained a T1-weighted coplanar image and a high-resolution T1-weighted anatomical volume (1 mm × 1 mm) acquired using a 3D-MPRAGE sequence.

### Preprocessing and analysis

Data were analyzed using FSL Version 5.0.2.2 FEAT Version 6.0^42^, MATLAB R2010B (MathWorks) and SPSS for Windows, Version 17.0, with visualization of neural results using MRIcron and MRICROGL^43^. Preprocessing of anatomical and functional images was performed in FSL, and consisted of skull-stripping using BET, removal of six volumes from the beginning of each functional run to ensure scanner stabilization, spatial smoothing with a Gaussian kernel of 8 mm FWHM, high-pass filtering at 40 s, and correction for interleaved slice timing. Functional runs were motion corrected using MCFLIRT. Motion parameters were low across all participants and were not included in the first-level design, with average absolute displacement = 0.13 voxel, min displacement = 0.01 voxel, max displacement = 0.47 voxel. Functional images were normalized using transforms estimated from individual subjects’ T1-weighted coplanar and high-resolution anatomical image (FLIRT: six degrees of freedom for registration to coplanar image, seven degrees for registration of the coplanar to the high-resolution image, and twelve degrees for registration to standard space). All coordinates are reported in MNI space. All reported neuroimaging main effects and contrasts use a standard cluster-corrected threshold of p < 0.05 (FSL, FEAT).

We specified a general linear model (GLM) with ten predictors, each convolved with a double-gamma hemodynamic response function. These regressors were #1 disgust priming, #2 neutral priming, #3 moral scenario reading, #4 non-moral scenario reading, #5 moral decisions following disgust priming, #6 moral decisions following neutral priming, #7 ‘acceptable’ responses, and #8 ‘unacceptable’ responses. Two nuisance regressors were also modeled, #9 for all button presses (4 per trial) and #10 for the gender classification task. Regressors #1 and #2 were of 1000 ms duration and locked to the onset of the facial prime, regressors #3–8 and #10 were all of variable length (locked to their respective onset and offsets in each trial), and regressor #9 was encoded as a fixed duration of 500 ms. Trials on which subjects did not make a response were simply excluded from analysis due to their low rate of occurrence (10 trials from 9 runs, across all participants). Of note, to facilitate the removal of nuisance variance without compromising our ability to examine our main regressors of interest we orthogonalized (using FSL’s orthogonalization options) the gender classification task regressor (#10) to each of the primes (#1 and #2), as well as the moral scenario reading (#3), non-moral scenario reading (#4), ‘acceptable’ responses (#7), and ‘unacceptable’ responses (#8) to both the disgust moral decisions (#5) and the neutral moral decisions (#6) regressors.

Examination of the main effects and contrasts of this model were aimed primarily to show replication of prior studies – that our task produces similar patterns of activations during moral judgment (regardless of prime condition) as those found in other studies.

Our first novel analysis was to examine which brain regions may account for the degree of change in moral judgment due to the priming manipulation. This analysis was accomplished through the application of an individual covariate analysis of the above-described model. Specifically, we examined which brain regions showed individual activation patterns at the time of disgust priming (regressor #1) that matched the individual behavioral effect of those primes on their moral acceptability ratings (disgust – neutral).

Based upon the regions identified by this covariate analysis and prior literature^44, 45^, we constructed a single region of interest (6 mm sphere; X:0, Y:38, Z:22; placed at the approximate centroid of a sub-cluster of voxels within the dmPFC). This region-of-interest (ROI), produced within MRIcron^43^, was utilized for a psychophysiological interaction (PPI) model to identify regions with functional connectivity to the dmPFC at the time of disgust priming (regressor #1). For the PPI analysis, the above model was re-run with two additional regressors – #11 the extracted timecourse of the ROI averaged across voxels and #12 the interaction between regressors #1 and #11 (i.e., multiplication of the two). The results of the PPI analysis led to production of another ROI within the left lateral occipital cortex, (6 mm sphere; X: −24, Y: −68, Z: 50; peak significant voxel).

To investigate how the emotionality of the prime altered the functional connectivity of this dmPFC ROI, we performed a second matched PPI analysis at the time of neutral priming (regressor #2). The results of this whole-brain analysis led to the construction of two large ROIs containing the voxels that showed significantly positive functional connectivity with the dmPFC ROI during the neutral priming condition.

Post-hoc, based upon recent separate findings from our lab which identified an adjacent region of the dmPFC to contain the information necessary to perform the transformation from objective value (count) to subjective utility (worth)^11^, we replicated our ROI analyses and PPI analyses using our daMCC ROI. We note that this daMCC ROI is non-overlapping with, and located just anterior and dorsal to our principal dmPFC ROI. The goal of these analyses was to attempt to define a potential mechanism through which moral judgments may be altered – through alterations in the determination of the subjective utility (worth) of the options being considered.

### Data Availability

The datasets generated during and/or analysed during the current study are not publicly available due to ongoing use, but are available from the corresponding author on reasonable request.
